# A Multi-Axis Framework for Late-Life Alzheimer’s Disease Interpretation

**DOI:** 10.3390/jpm16030157

**Published:** 2026-03-10

**Authors:** Yong Tae Kwak, YoungSoon Yang

**Affiliations:** 1 Department of Neurology, Hyoja Geriatric Hospital, 1-30, Jungbu-daero 874beon-gil, Giheung-gu, Yongin-si 17089, Gyeonggi-do, Republic of Korea; 2 Department of Neurology, Soonchunhyang University Cheonan Hospital, 31 Soonchunhyang 6-gil, Dongnam-gu, Cheonan-si 31151, Chungcheongnam-do, Republic of Korea; astro76@naver.com

**Keywords:** Alzheimer’s disease, amyloid PET, amyloid-β oligomers, MDS-OAβ (multimer detection system), postoperative delirium

## Abstract

Late-life Alzheimer’s disease (AD) is increasingly defined by biomarkers, yet in adults aged ≥65 years the relationship between amyloid positivity and near-term cognitive decline is often discordant. Amyloid PET robustly detects fibrillar plaque burden, but it incompletely captures dynamic and potentially neurotoxic amyloid processes, particularly soluble assemblies and oligomer-related “activity.” This review rethinks the late-life AD spectrum by integrating four clinical lenses that frequently drive real-world interpretive uncertainty: (1) amyloid PET positivity as a measure of fibrillar plaque presence and magnitude; (2) plasma amyloid-β oligomerization tendency measured by the multimer detection system (MDS-OAβ) as an activity-oriented (i.e., a dynamic readout hypothesized to reflect ongoing processes rather than cumulative lesion burden), process-linked readout that may decouple from plaque burden; (3) postoperative delirium (POD) as a time-anchored stress-test phenotype revealing vulnerability and reduced resilience under systemic insults; and (4) drug-linked biomarker trajectories, contrasting rapid plaque removal by anti-amyloid monoclonal antibodies with observational signals raising the hypothesis that Ginkgo biloba may be associated with oligomer-related biology and, in some contexts, differences in longitudinal amyloid accumulation trajectories in the absence of observed immediate plaque reduction. We propose a pragmatic multi-axis framework—plaque burden, amyloid activity, downstream engagement, and vulnerability/resilience—to contextualize late-life discordances such as PET positivity without decline, PET negativity with elevated MDS-OAβ, delirium-associated decompensation, and clinical change without rapid PET decline. This synthesis highlights testable predictions and prioritizes longitudinal, multi-marker studies to determine whether activity-oriented biomarkers and stress phenotypes can refine late-life risk stratification beyond plaque-centered models.

## 1. Introduction

Alzheimer’s disease (AD) is now widely conceptualized as a biological continuum rather than a purely clinical syndrome. This shift was formalized by the NIA–AA Research Framework, which defines AD in research settings according to biomarkers of amyloid deposition (A), tau pathology (T), and neurodegeneration or neuronal injury (N), independent of clinical symptoms [1,2]. More recently, the Alzheimer’s Association Workgroup updated the 2018 NIA-AA research framework by proposing revised criteria for the diagnosis and staging of Alzheimer’s disease, aiming to bridge research and clinical care by incorporating advances in biomarker assessment (including fluid-based biomarkers) and providing an updated biologic diagnosis and staging scheme [3]. This biology-first approach has provided an essential foundation for therapeutic development, trial enrichment, and mechanistic clarity. However, its interpretive limits become increasingly evident in late life, where AD-related biology becomes common while dementia remains heterogeneous and far from inevitable.

In older adults, amyloid PET positivity frequently coexists with preserved cognitive function for prolonged periods, and rates of decline vary widely even among individuals with similar biomarker profiles [4,5]. Such late-life discordances are not merely statistical noise. Rather, they reflect the convergence of multiple age-dependent processes—including amyloid and tau pathology, cerebrovascular disease, systemic frailty, inflammatory states, and resilience factors—whose timing and relative dominance differ substantially across individuals. Complementing plaque-centric interpretations, late-life AD has also been framed as, at least in part, a brain expression of systemic metabolic dysfunction. In this view, cardiometabolic stressors (e.g., insulin resistance, oxidative stress, and inflammatory signaling) may shape vulnerability/reserve and contribute to symptom expression and short-term trajectory variability even when fibrillar amyloid PET burden is relatively stable [6,7]. As a result, single biomarker-defined AD in late life often fails to map cleanly onto short-term clinical trajectories.

The present review therefore adopts a late-life focus grounded in empirical observation rather than theoretical redefinition. In this context, late-life Alzheimer’s disease refers to Alzheimer-spectrum biology, defined as the presence of AD-related pathological processes—most commonly amyloid positivity—across the clinical continuum, in adults aged 65 years and older. At this stage, pathological markers become increasingly prevalent, yet clinical trajectories remain highly variable and frequently discordant from biomarker status. In late life, Alzheimer-related biology intersects with aging-related vulnerability, acute physiological or cognitive stressors, and heterogeneous resilience, complicating linear interpretations that link static biomarkers directly to near-term cognitive outcomes.

This perspective emerged from a series of studies conducted by our group in older adults over 65 years, spanning perioperative cohorts as well as biomarker-confirmed Alzheimer’s disease and mild cognitive impairment. Across these investigations, plasma amyloid-β oligomerization tendency (MDS-OAβ) repeatedly appeared as a convergent biological signal linking acute cognitive vulnerability—most notably postoperative delirium—to longer-term clinical trajectories, even when amyloid PET findings were stable or discordant [8,9]. Complementary studies in amyloid PET-positive AD and mild cognitive impairment (MCI) further suggested that oligomer-related biology may be modifiable, with clinical and functional changes observed in the absence of measurable plaque reduction [10,11]. In addition, our group has conducted two related analyses: (i) a large perioperative cohort study evaluating pre- and postoperative plasma MDS-OAβ for postoperative delirium risk stratification (currently under peer review); and (ii) a longitudinal amyloid-positive MCI study examining associations between Ginkgo biloba use and trajectories of amyloid PET burden and plasma Aβ oligomerization tendency (accepted for publication). These studies are referenced for context and hypothesis generation and should not be interpreted as primary evidentiary support for the framework.

Taken together, these observations motivated the present review. Rather than proposing a new disease subtype, we use the term late-life AD to emphasize an interpretive framework—one that recognizes Alzheimer-related biology as necessary but not sufficient to explain cognitive outcomes in older adults. Within this framework, late-life discordances—such as amyloid PET positivity without near-term decline, elevated oligomer-related biomarkers in clinically normal individuals, or clinical improvement without amyloid plaque removal—are viewed not as anomalies, but as characteristic features of Alzheimer’s disease as it manifests in aging brains.

Against this background, we propose a reinterpretation of late-life AD through four clinical lenses that reflect where clinical interpretation most commonly diverges in real-world practice. Rather than constituting a taxonomy derived from individual studies, each lens corresponds to a distinct and non-reducible clinical question encountered in the care of older adults with suspected or established Alzheimer-spectrum disease. (1) Amyloid PET positivity primarily addresses whether fibrillar amyloid pathology is present; (2) plasma MDS-OAβ provides a dynamic readout of amyloid oligomerization tendency and an activity-like peripheral signal (with unknown specificity for CNS oligomer burden), which may precede or decouple from plaque accumulation; (3) postoperative delirium functions as an acute stress-test phenotype, implicitly probing the brain’s vulnerability to physiological or cognitive insults; and (4) drug-linked biomarker trajectories shift the focus from static diagnosis to biological response, contrasting plaque-removing anti-amyloid monoclonal antibodies with signals from biomarker-defined cohorts suggesting that Ginkgo biloba extract may be associated with oligomer-related peripheral biomarker patterns and, in some contexts, hypothesized differences in amyloid biomarker trajectories in observational settings. The proposed lenses are conceptually complementary to AT(N) while addressing dimensions that are only partly captured by it. Amyloid PET primarily reflects ‘A’ (fibrillar plaque burden), whereas downstream engagement may map to ‘T/N’. Vulnerability/reserve—operationalized here by stress-test phenotypes such as postoperative delirium—represents an additional axis relevant to short-term trajectories in late life.

The purpose of this framework is not to replace established biomarker models, but to provide a pragmatic interpretive scaffold for late-life discordances—such as amyloid PET positivity without near-term decline, PET negativity with elevated MDS-OAβ, or clinical improvement without plaque reduction—by situating them as different positions along interacting biological and clinical trajectories that are particularly salient in late life. Although tau biomarkers (e.g., plasma or CSF phosphorylated tau) represent a critical axis in Alzheimer’s disease biology, their routine clinical availability and accumulated real-world experience—particularly in blood-based assays—remain limited in current practice; therefore, detailed discussion of tau biomarkers is beyond the scope of the present review. In future work, tau biomarkers—particularly plasma p-tau species (e.g., p-tau217/p-tau231) and related markers such as NfL/GFAP—could be incorporated as an additional axis or as a modifier of downstream engagement to further refine trajectory interpretation as clinical availability matures; conceptually, PET−/high p-tau and PET+/low p-tau would represent testable discordant states expected to diverge longitudinally in symptom coupling and downstream marker trajectories. Because parts of this framework were motivated by observations from our biomarker-defined and perioperative cohorts, we present selected author-led findings as illustrative, hypothesis-generating examples rather than definitive evidence of efficacy or causality. We therefore emphasize independent and multicenter studies (including mixed or negative results) and highlight the need for replication and longitudinal validation across diverse cohorts.

1. What amyloid PET measures in late life—and what it cannot

Amyloid PET is one of the most robust in vivo tools for detecting fibrillar amyloid-β plaque deposition. Its central value is that it quantifies an accumulated burden that is otherwise accessible only at autopsy, enabling biologically anchored diagnosis, staging, and trial enrichment. Quantification has become more standardized through the Centiloid approach, which scales amyloid PET measurements across tracers and analytic pipelines onto a common 0–100 metric [12]. Centiloid thresholding has also been examined for predicting long-term progression risk in some settings [13]. Standardization strengthens longitudinal interpretation and improves cross-study comparability. It also supports a more clinically meaningful view of amyloid burden as a continuous variable rather than a binary label.

However, amyloid PET is not a direct readout of the entire amyloid system. It largely reflects fibrillar plaque, not soluble oligomeric assemblies, and it does not directly measure synaptic dysfunction, neuroinflammation, blood–brain barrier integrity, tau propagation, or vascular brain injury. These distinctions matter more in late life than they do in younger cohorts because older adults commonly have competing neurodegenerative and vascular processes, along with systemic factors that can acutely influence cognition. In an older patient, plaques may represent a long-standing biological substrate while the proximate causes of deterioration may be delirium, medication burden, sleep disruption, infection, metabolic instability, or vascular decompensation. Therefore, amyloid PET answers a crucial question—how much fibrillar plaque is present—but it does not, by itself, answer the late-life questions that families most often ask: why is decline happening now, and what will happen next?

This is precisely why binary interpretations are frequently brittle in older adults. “PET positive” can coexist with stable cognition, and “PET negative” can coexist with high vulnerability. The updated appropriate use criteria (AUC) for amyloid and tau PET emphasize that PET should be deployed in clinically appropriate scenarios and interpreted within comprehensive clinical evaluation rather than as a standalone adjudicator [14]. This guidance aligns with what the late-life clinic repeatedly teaches: interpretation should focus on axes and trajectories rather than labels.

2. How common is amyloid positivity in cognitively unimpaired older adults?

The interpretive meaning of amyloid positivity changes with age partly because the population prevalence changes with age. A large individual participant data meta-analysis demonstrated that the prevalence of amyloid pathology increases substantially with age even among individuals without dementia [4]. More recent work also provides prevalence estimates of amyloid abnormality across the Alzheimer disease clinical spectrum [15]. In practical terms, this means that a positive amyloid PET scan in an 80-year-old does not automatically have the same explanatory weight as the same scan in a 55-year-old with progressive amnestic symptoms. In late life, amyloid positivity may indicate biological AD, but it can also indicate that the individual is a plaque carrier within a mixed-pathology environment where reserve and competing conditions shape symptoms. Thresholds further complicate interpretation. PET is often dichotomized into positive/negative categories, but clinically meaningful thresholds vary by purpose: diagnostic classification, prediction of future accumulation, or trial eligibility [16]. Work focusing on lower burdens suggests that relatively low amyloid PET levels can predict future accumulation and cognitive decline in certain populations, supporting the concept of subthreshold or “early positive” states [17]. In late life, thresholds should therefore be treated as tools for probabilistic risk communication rather than deterministic labels.

3. From a single-axis cascade to interacting trajectories

The classic AD cascade model—amyloid accumulation followed by tau spread, neurodegeneration, and clinical decline—remains useful, especially in early symptomatic disease [18]. Late-life trajectories, however, often violate the simplicity of a single-axis cascade. Some older adults show high plaque burden with slow clinical change, while others experience rapid decline with modest plaque signals but strong vascular injury, inflammation, frailty, or recurrent stressors [19,20]. Rather than abandoning the cascade, it is more faithful to treat it as one thread within a web of interacting processes. Downstream markers illustrate why the web matters. Tau pathology correlates more closely with symptoms than plaques, and blood-based phosphorylated tau markers increasingly capture amyloid-linked downstream changes and help differentiate AD from other neurodegenerative disorders [21,22]. Meanwhile, sleep and systemic physiology influence vulnerability: experimental work has demonstrated that sleep facilitates metabolite clearance in the brain [23], and human experimental/clinical studies link sleep disruption with amyloid biology [24,25]. These considerations support a practical late-life view in which processes operate at different timescales: slow plaque accumulation, intermediate tau/neurodegeneration evolution, and fast vulnerability shifts triggered by stressors. A single biomarker snapshot cannot capture all three timescales.

4. Why MDS-OAβ reframes “amyloid activity” in late life

In late-life practice, clinicians often know plaque burden but still face questions about activity: is amyloid-related biology dynamically contributing to current vulnerability, or is plaque burden a static background? Plasma MDS-OAβ is one candidate approach to address this gap. MDS-OAβ is described as a plasma-based measure of the tendency of plasma conditions to promote amyloid-β oligomer formation under assay conditions [26]. The conceptual attraction of oligomer-related markers is longstanding: soluble oligomeric assemblies are frequently considered more synaptotoxic than fibrillar plaque, and they may vary by disease stage in ways plaque burden cannot capture [27]. Blood oligomerization-based approaches have also been linked to amyloid PET positivity in some cohorts [28]. In blinded validation work, blood amyloid-β oligomerization has been reported to discriminate AD from controls in defined samples [29]. Additional plasma oligomer studies support clinical associations with severity and cerebral amyloid deposition [30]. Earlier work also proposed oligomeric forms in plasma as a potential blood-based biomarker conceptually aligned with this direction [31].

5. Why oligomers matter: bridging symptoms and vulnerability

Oligomers occupy a complicated but important place in AD biology. They are often framed as soluble assemblies that can interfere with synaptic function and potentially trigger downstream pathology [32], and emerging evidence suggests oligomer-related signals may peak early along the AD continuum and precede overt tau pathology in some biomarker-defined samples [33]. If this pattern is correct, it implies that oligomer-related markers can behave like stage-sensitive signals that do not simply mirror plaque burden. This matters in late life because older brains have reduced physiological reserve. Small perturbations in synaptic efficiency, inflammatory tone, cerebral perfusion, or sleep architecture can produce outsized clinical effects. Thus, even when plaque burden is stable, a dynamic oligomer-related signal—if validated—could help explain why symptoms worsen with intercurrent illness, hospitalization, anesthesia exposure, pain, or medication changes. This does not require assuming oligomers are the sole driver of symptoms. It requires only acknowledging that plaque burden and synaptic stress may not move in lockstep, particularly in older adults navigating multiple comorbidities.

6. What MDS-OAβ measures: a cautious functional interpretation

MDS-OAβ should be interpreted cautiously as a measure of plasma conditions influencing amyloid-β oligomer formation in the assay rather than as a direct quantitative measurement of brain oligomer concentration. Accordingly, throughout this review we treat plasma MDS-OAβ as a context-dependent dual-use marker—a peripheral ‘activity-like’ readout of oligomerization tendency and a systemic vulnerability phenotype—whose clinical meaning is best inferred from longitudinal trajectory and stress context (e.g., perioperative vulnerability), rather than as a direct surrogate for CNS oligomer burden (see Supplementary Box S1). In addition, genetic susceptibility (e.g., APOE ε4) may modulate oligomer dynamics and clearance across brain and peripheral compartments and has been associated with plasma/CSF oligomer patterns and MDS-OAβ–related signals. This is both a limitation and strength. It is a limitation because peripheral factors—protein binding, inflammation/acute illness, renal or hepatic function, frailty-related metabolic state—may influence signal, complicating simple inference about the brain. It is a strength because those systemic and genetic factors are precisely what shape late-life vulnerability. Late-life outcomes are shaped by brain–body coupling; therefore, peripheral dynamic signals can carry clinically meaningful information even when they are not one-to-one proxies for a single brain compartment. A pragmatic late-life use of MDS-OAβ is therefore pattern-based rather than label-based. Does the signal persist or fluctuate? Does it couple with vulnerability events? Does it align with downstream markers such as plasma p-tau? Does it predict clinical trajectories in biomarker-defined cohorts? This approach aligns with the reality that older adults often cannot be responsibly interpreted from a single timepoint result.

7. Evidence linking MDS-OAβ to Alzheimer-spectrum biology

Evidence supporting MDS-OAβ as an Alzheimer-spectrum biomarker includes methodological work demonstrating dynamic plasma oligomer signals [26], and blinded validation suggesting discriminative utility in defined samples [29]. Beyond assay feasibility, the key late-life need is to evaluate MDS-OAβ in biologically defined, ideally multicenter cohorts against clinically meaningful longitudinal outcomes, given potential variability across samples and assay conditions. As an illustrative, hypothesis-generating example in such a biologically constrained setting, we examined MDS-OAβ trajectories alongside longitudinal clinical outcomes in our cohorts. In amyloid PET-positive AD patients treated with donepezil, adjunctive Ginkgo biloba was associated with improved cognitive outcomes and greater decreases in plasma MDS-OAβ compared with donepezil alone in our cohort [10]. In amyloid PET-positive MCI patients, Ginkgo monotherapy was similarly associated with preserved cognition and reduced plasma oligomerization tendency [11]. These findings should not be overstated as definitive efficacy claims; rather, they illustrate a perspective-relevant point: in biomarker-defined late-life cohorts, dynamic oligomer-related trajectories can be studied alongside clinical outcomes, and changes in MDS-OAβ can accompany changes in symptoms. This supports the broader thesis that plaque burden and activity-like signals can diverge and that the divergence itself may be clinically informative. A concise summary distinguishing measurement scope and interpretive levels for MDS-OAβ is provided in Supplementary Box S1.

8. The key discordance—PET negative but MDS-OAβ high—and how to adjudicate it

One of the most challenging late-life patterns is discordance between amyloid PET and MDS-OAβ, particularly PET negative with high MDS-OAβ. It is tempting to force a binary resolution—either the blood-based signal is wrong or the PET missed amyloid. Late life rarely rewards such forced dichotomies. A more faithful approach treats discordance as a longitudinal question. PET negativity lowers the probability of high fibrillar plaque burden; it does not eliminate the possibility of early/subthreshold amyloid biology, nor does it exclude systemic drivers that influence the plasma signal. The responsible adjudication strategy is therefore pattern-based: repeat measurement, assess clinical trajectory, evaluate downstream markers where appropriate, and reconsider imaging if the course demands it. In late life, the primary function of an activity-like signal is often to prevent false reassurance. A negative PET scan may lower the probability that plaques are the dominant substrate, but it does not guarantee stability in an older adult with substantial vulnerability [34]. If a dynamic plasma signal remains persistently high and the individual experiences stress-test failure, the prudent conclusion is not “non-AD” but “high vulnerability with uncertain dominant substrate,” warranting careful longitudinal follow-up.

9. POD as a window into Alzheimer-spectrum vulnerability

POD represents a time-anchored clinical event that reliably exposes late-life brain vulnerability and predicts adverse long-term cognitive outcomes. Rather than being a purely transient behavioral syndrome, delirium frequently reflects failure of cerebral reserve under physiological stress and can unmask previously compensated cognitive impairment. Multiple longitudinal studies have shown that POD is associated with subsequent cognitive decline, positioning it as a meaningful inflection point along aging and Alzheimer-related trajectories [35,36,37]. In aging societies where surgery and hospitalization are common, POD therefore constitutes one of the most practical and reproducible stress tests of late-life brain resilience. From a biomarker perspective, POD is uniquely informative because it creates a defined before–after window in which stress-response biology can be observed. This contrasts with slowly progressive cognitive decline, where temporal coupling between biological change and clinical manifestation is often ambiguous. If Alzheimer-spectrum processes contribute to vulnerability, their influence may become most apparent when the system is acutely perturbed—as occurs during surgery and anesthesia—yet POD remains multifactorial and is also driven by perioperative factors such as anticholinergic burden, hypoxia, infection/inflammation, pain, and anesthesia depth; accordingly, this framework does not imply amyloid primacy but contextual relevance in a subset. Mechanistically, human studies increasingly implicate dynamic processes rather than fixed structural injury. Prospective cohort work has linked POD to blood–brain barrier (BBB) dysfunction [38], and other cohorts have demonstrated coupling between POD, BBB breakdown, neuroinflammatory responses, and CSF lactate shifts—pointing to a stress-responsive metabolic–inflammatory physiology rather than a plaque-driven mechanism [39]. These pathways are particularly relevant to Alzheimer-spectrum interpretation because BBB dysfunction and inflammation can amplify downstream neurodegenerative cascades and transiently worsen cognition, even in the absence of changes in plaque burden. Viewed through a multi-axis framework, POD-related mechanisms primarily map onto the vulnerability/reserve axis. They can operate independently of amyloid plaque load, contribute to symptom expression when plaque burden is stable, and accelerate decline by lowering physiological reserve. Interpreting delirium solely through a plaque-centric lens is therefore biologically incomplete; evidence from multiple perioperative cohorts instead supports stress-responsive, non-plaque pathways (e.g., BBB dysfunction, neuroinflammation, metabolic shifts) as clinically actionable dimensions of late-life brain vulnerability

10. Why amyloid plaque-centric biomarkers fail for POD, and what oligomer-related signals add

Historically, attempts to link AD biomarkers to delirium risk have focused on amyloid-centric biomarkers, such as CSF Aβ42 (often in combination with tau measures), along with tau and plasma monomeric Aβ. Results have been inconsistent across cohorts. One illustrative example is a prospective study reporting that a preoperative CSF β-amyloid/tau ratio was associated with POD risk, supporting the idea that pre-existing AD-related vulnerability can influence delirium risk, yet not reducing delirium to plaque burden alone [40]. In parallel, plasma monomeric Aβ has generally shown poor predictive performance in perioperative settings. A representative prospective study reported that perioperative changes in plasma Aβ were not strongly associated with POD, highlighting a broader limitation of monomeric plasma measures in capturing the biology underlying acute network dysfunction [41]. This pattern of negative or inconsistent findings suggests a conceptual mismatch. Delirium is best understood as failure of synaptic and network function under acute physiological stress, rather than as a direct consequence of cumulative plaque burden. Biomarkers optimized to index long-term amyloid deposition may therefore be poorly suited to detect vulnerability to short-term stressors. If delirium reflects stress-induced network fragility, amyloid species more closely linked to synaptic toxicity—such as oligomer-related assemblies—may provide more relevant biological information than plaque load or monomer concentration. Emerging perioperative evidence supports this shift in focus. In an initial prospective observational study, postoperative plasma amyloid-β oligomerization tendency (MDS-OAβ) was higher in patients who developed postoperative delirium than in those who did not, and postoperative MDS-OAβ levels were correlated with delirium severity [8]. A subsequent prospective pilot study confirmed that elevated preoperative MDS-OAβ was associated with both the occurrence and severity of POD and that pre- and postoperative values were highly correlated, consistent with a predominantly trait-like vulnerability rather than a transient surgical artifact [9]. In a more recent and substantially larger prospective cohort (preliminary; under peer review), preoperative MDS-OAβ showed promising discrimination in this sample for POD, remained associated with delirium severity, and showed limited short-term perioperative change, with an exception observed among patients who developed delirium. Together, these findings support the interpretation of MDS-OAβ as indexing an underlying vulnerability that lowers the threshold for delirium when exposed to perioperative stress. Importantly, these data should be interpreted as supporting risk stratification rather than definitive causal inference. They require replication in multicenter cohorts with careful control for confounders such as frailty, vascular burden, medication exposure, and perioperative complications. Moreover, even if future work demonstrates that plasma oligomer-related signals are only indirectly related to brain oligomer burden, their association with POD would remain clinically meaningful, given the central role of brain–body coupling in late-life outcomes. Within a late-life Alzheimer-spectrum framework, these observations suggest that oligomer-related plasma signals capture a dimension of stress sensitivity that is not redundant with amyloid PET. POD may therefore serve as a biologically informative stress test that helps adjudicate discordant biomarker profiles. Elevated MDS-OAβ in PET-negative individuals who develop POD may reflect an active vulnerable state rather than a false-positive signal, whereas PET-positive individuals with low MDS-OAβ who remain delirium-free may represent a resilient or “silent amyloid” phenotype. In this way, oligomer-related measures complement plaque-centric biomarkers by providing functional insight into vulnerability, rather than duplicating information about cumulative amyloid burden.

11. Why the “activity” lens matters for therapy interpretation and why trajectories must be compared on multiple axes

In late life, the activity lens matters because it changes how we interpret therapeutic biomarker trajectories. Plaque-lowering monoclonal antibodies demonstrate that amyloid PET can show substantial target engagement while clinical effects remain modest and heterogeneous [42,43]. This should not be read as failure. It should be read as confirmation that plaque burden is one axis among several, and that late-life trajectories can be dominated by vulnerability amplification or non-AD pathology. If an older patient has high frailty burden, significant vascular disease, or recurrent stressors, plaque reduction may not translate into proportionate symptomatic benefit. Conversely, interventions that do not directly clear plaques might still influence dynamic signals and symptoms through mechanisms such as oxidative stress modulation, vascular effects, sleep stabilization, or inflammation modulation, without dramatically altering PET signals. This makes longitudinal multi-axis tracking essential: plaque burden, downstream tau markers, neurodegeneration indices, and vulnerability phenotypes should be compared rather than assumed to align. The updated appropriate use criteria reinforce that PET should be interpreted within comprehensive clinical evaluation and evolving therapeutic contexts [14]. Importantly, late-life Alzheimer-spectrum presentations are commonly embedded in a comorbidity-rich context that can modify clinical trajectories and biomarker–symptom relationships. Co-occurring conditions—including sleep disorders, chronic pain/inflammation, neuropsychiatric syndromes, and medication effects (e.g., anticholinergic burden), as well as systemic illness—may amplify vulnerability or mimic/compound cognitive decline independent of plaque burden. Accordingly, this framework emphasizes explicit comorbidity/reserve profiling and longitudinal confirmation when adjudicating discordant profiles [44].

12. Drug-linked biomarker trajectories: anti-amyloid monoclonal antibodies versus ginkgo in a biomarker-defined framework

**Figure 1:**
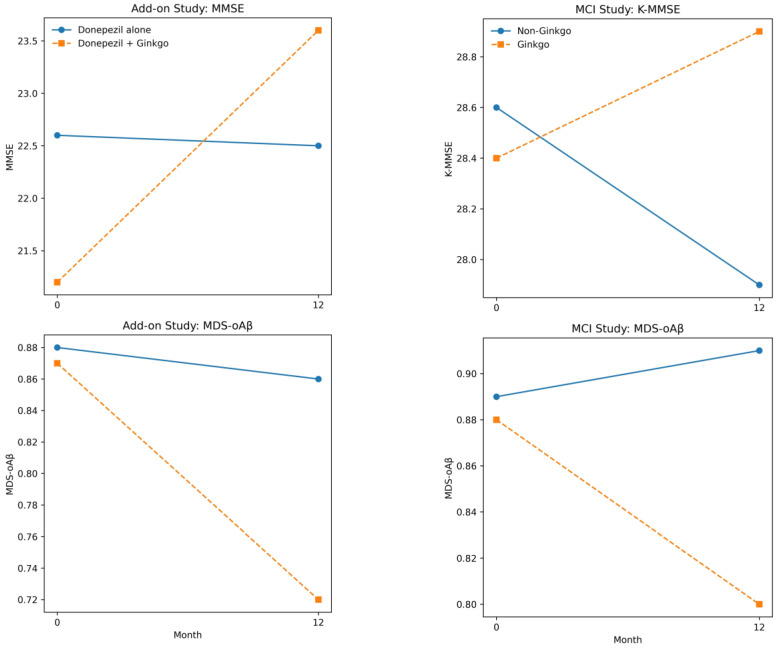
Effects of Ginkgo biloba on cognitive performance and plasma oligomerized amyloid-β over 12 months in different clinical contexts. The **left panels** present the add-on study evaluating the effect of Ginkgo biloba when added to donepezil treatment, while the **right panels** present the mild cognitive impairment (MCI) cohort comparing patients treated with and without Ginkgo biloba. **Upper panels** show changes in MMSE, and **lower panels** show changes in plasma oligomerized amyloid-β (MDS-oAβ). Solid lines indicate non-Ginkgo control groups (donepezil alone or non-Ginkgo), and dashed lines indicate Ginkgo-treated groups. The figure was newly generated based on previously published data.

Drug-linked biomarker trajectories illustrate the interpretive value of a biomarker-defined, multi-axis approach. For monoclonal antibodies targeting amyloid, amyloid PET provides a direct measure of the therapy’s intended biological effect and helps contextualize risk–benefit decisions [42,43]. For adjunctive or alternative strategies such as Ginkgo biloba, evaluation should not be reduced to plaque reduction alone, particularly when hypothesized mechanisms include synaptic modulation, antioxidant effects, vascular modulation, or broader resilience pathways. At the population level, systematic reviews and meta-analyses have reported variable efficacy signals of Ginkgo biloba in dementia, emphasizing heterogeneity across clinical contexts [45,46]. Large prevention trials have also reported mixed outcomes in older populations [47,48]. These broader results motivate a more biologically anchored question: in amyloid-confirmed late-life cohorts, do clinical trajectories align with changes in an activity-like biomarker axis even when plaque is unchanged? In our biomarker-defined cohorts restricted to amyloid PET-positive individuals, adjunctive Ginkgo biloba to donepezil was associated with improved cognitive outcomes and decreased plasma MDS-OAβ relative to donepezil alone [10], and Ginkgo monotherapy in amyloid PET-positive MCI was associated with preserved cognition and reduced MDS-OAβ [11] (Figure 1). In our recent 18-month extension analysis (preliminary; under peer review), baseline MDS-OAβ levels and amyloid PET burden were jointly associated with subsequent clinical trajectories, with continued Ginkgo use being linked to sustained clinical improvement. Although Ginkgo treatment was not associated with a reduction in amyloid PET burden over time, amyloid PET signal showed a progressive increase in the non-Ginkgo group, which was closely coupled with longitudinal increases in MDS-OAβ. Together, these observations suggest an apparent dissociation between amyloid plaque accumulation and plasma oligomer-related trajectories; however, causality cannot be inferred, this pattern may reflect residual confounding, and it should be interpreted as hypothesis-generating. Importantly, absence of plaque reduction does not, by itself, establish disease modification (or its absence). So, these findings should be framed as perspective-generating rather than definitive efficacy claims. Their relevance here is conceptual: selecting a biologically homogeneous cohort (PET-positive) helps clarify whether clinical trajectories align with changes in an activity-like biomarker. The broader lesson is that biomarker trajectories are not interchangeable. A drug can lower plaques without fully correcting vulnerability, and an intervention can shift dynamic biology and symptoms without large plaque changes. Definitive testing would require a randomized, biomarker-stratified trial using a standardized extract/dose, with adherence verification, safety monitoring, and clinically meaningful endpoints beyond MMSE. A multi-axis framework reduces surrogate confusion and helps define what should be measured for each mechanism.

13. Integrative late-life model—silent amyloid, active amyloid, vulnerability amplification—and what it predicts

**Figure 2:**
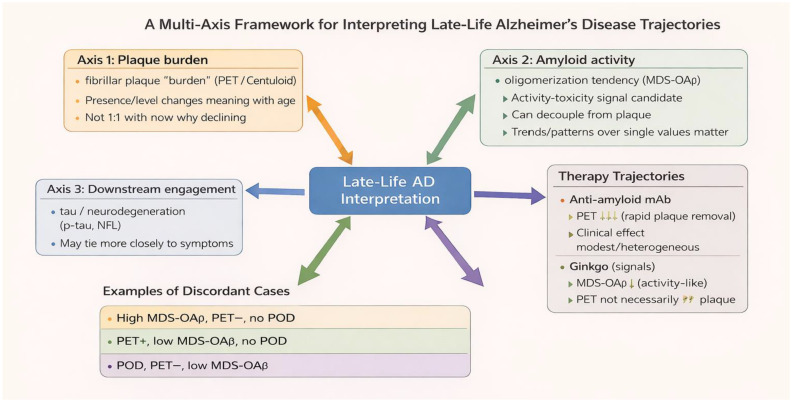
A Multi-Axis Framework for Interpreting Late-Life Alzheimer’s Disease Trajectories. The schematic highlights three simplified discordant patterns (from Table 1): high MDS-OAβ with PET−/POD−, PET+ with MDS-OAβ−/POD− (“silent amyloid”), and POD+ with PET−/MDS-OAβ− (event-dominant vulnerability). The framework emphasizes longitudinal, multi-marker interpretation and cautions that biomarker changes may reflect different axes depending on the intervention.

Putting these elements together, we propose a practical late-life model with three interpretable states that can coexist and transition: silent amyloid burden, active/dynamic amyloid-related biomarker state, and vulnerability amplification (Table 1, Figure 2). “Silent amyloid” refers to individuals with amyloid PET positivity whose near-term clinical trajectory is dominated by reserve and competing pathologies; plaques function as background risk. “Active/dynamic amyloid-related biomarker state” refers to states where dynamic signals (candidate markers such as MDS-OAβ and downstream p-tau measures) suggest ongoing Alzheimer-spectrum pathophysiology that is more tightly coupled to symptoms. “Vulnerability amplification” refers to states where stress-test phenotypes such as POD reveal low reserve and heightened sensitivity to systemic perturbation, often mediated through BBB dysfunction and inflammation [38,39]. This model generates clinically useful predictions. First, late-life variance should be reduced when biomarkers are interpreted longitudinally rather than as a one-time label. Second, stress-test failures such as POD should identify a subgroup at risk for accelerated decline regardless of baseline plaque status, because vulnerability mechanisms can dominate. Third, therapy response heterogeneity should map partly onto axis alignment: individuals whose symptoms are tightly coupled to Alzheimer-spectrum dynamic biology may show more symptomatic benefit from amyloid-directed interventions than individuals whose trajectories are dominated by vulnerability or non-AD pathology. Importantly, these predictions are testable in longitudinal, biomarker-enriched cohorts; key designs and falsification criteria are summarized in Box 1. Until then, the model functions as an interpretive guardrail: it discourages over-labeling based on plaques alone, discourages false reassurance from a single negative biomarker, and discourages treating any biomarker change as a universal surrogate for clinical benefit.

Box 1 **Testable Predictions and Study Designs for the Multi-Axis Framework.**

**Falsifiable predictions (examples)**

- Discordance subgroup prediction: PET−/high MDS-OAβ individuals will show higher risk of subsequent cognitive decline than PET−/low MDS-OAβ after adjustment for age, education/reserve, and comorbidities.
- Stress-test interaction prediction: In perioperative cohorts, POD will magnify short-term cognitive worsening and accelerate longer-term decline most strongly in those with elevated vulnerability/reserve burden, regardless of baseline PET status.
- Axis-specific trajectory prediction: In PET+ biomarker-defined cohorts, within-person changes in MDS-OAβ will track symptom trajectories more closely than changes in fibrillar plaque measures, consistent with dissociable ‘burden’ vs. ‘activity-like’ signals.

**Suggested study designs**

- Prospective perioperative cohorts with pre- and post-event sampling (MDS-OAβ ± plasma p-tau/NfL), standardized delirium ascertainment, and multi-year cognitive follow-up.
- Biomarker-enriched PET-defined MCI/early AD samples with repeated plasma measures and longitudinal outcomes to test whether trajectory alignment predicts response heterogeneity.
- Multicenter replication using harmonized pre-analytical handling and assay protocols to evaluate robustness and generalizability.

**Short-term vs. long-term expectations (hypotheses to be tested)**

- Short-term outcomes: vulnerability/reserve and stress-response biology are expected to dominate (e.g., POD, intercurrent illness, frailty-related decompensation).
- Long-term outcomes: cumulative burden and downstream engagement are expected to contribute more strongly, with interactions across axes shaping heterogeneity.

Testable predictions and feasible study designs derived from the proposed multi-axis framework. Predictions are intended to be falsifiable and hypothesis-generating rather than definitive estimates and should be evaluated in independent, multicenter cohorts with standardized pre-analytical handling. Abbreviations: AD, Alzheimer’s disease; MCI, mild cognitive impairment; PET, positron emission tomography; POD, postoperative delirium; MDS-OAβ, plasma oligomerization tendency assay; p-tau, phosphorylated tau; NfL, neurofilament light chain.

14. Subclinical Amyloid Vulnerability, POD, and the Potential Preventive Role of Ginkgo biloba

In our previous perioperative cohort of cognitively normal older adults with no evidence of cognitive impairment prior to surgery, a descriptive estimate derived from the raw baseline MDS-OAβ data (not reported in the published POD-focused article [8]) indicated that 42.3% (44/104) of participants exceeded the AD diagnostic cut-off of 0.78, suggesting that elevated values were already prevalent in this population. Consistent with this observation, preliminary findings from an independent cohort—currently under peer review and not yet published—suggest that approximately 35% of cognitively normal older adults may exceed the same threshold; this estimate is provided for context only. These findings suggest that biomarker-defined amyloid-related vulnerability is common even among individuals considered clinically normal. This subclinical vulnerability appears to have clinically meaningful correlates. The high prevalence of elevated MDS-OAβ in cognitively normal older adults and its association with POD support the possibility that a subset of these individuals may lie within a very early or preclinical segment of the AD spectrum. In this context, additional evidence from amyloid PET-positive MCI and AD provide a relevant point of comparison. In our studies, Ginkgo biloba treatment in these populations was associated with more favorable cognitive trajectories alongside reductions in MDS-OAβ [10,11]. Moreover, in an 18-month extension analysis (unpublished; under peer review), continued Ginkgo use was associated with sustained clinical stability, stable amyloid PET standardized uptake value ratios, and a decrease in MDS-OAβ, whereas non-treated individuals showed progressive increases in amyloid PET burden accompanied by rising MDS-OAβ levels. Although these findings do not demonstrate a plaque-reducing effect of Ginkgo biloba, the close coupling between MDS-OAβ trajectories and clinical outcomes across POD, MCI, and AD populations raises the hypothesis that modulation of oligomer-related amyloid toxicity may be clinically relevant. Consequently, these observations motivate a testable hypothesis: cognitively normal older adults with persistently elevated MDS-OAβ—particularly those who manifest POD under physiological stress—may represent an enriched vulnerability subgroup for prospective follow-up studies. Such a research approach remains hypothesis-generating and requires independent prospective validation, but it highlights the potential role of biomarker-guided risk stratification beyond symptom-based definitions of normal aging. Key falsifiable predictions and feasible study designs derived from this model are summarized in Box 1.

## 2. Conclusions

Late-life Alzheimer-spectrum interpretation is best treated as a triangulation task across burden, activity-like biology, and vulnerability/reserve rather than as a single-axis binary decision. Amyloid PET remains indispensable for measuring fibrillar plaque burden and for documenting target engagement in plaque-lowering therapies. Yet late-life heterogeneity means plaque burden alone often cannot explain symptom timing, short-term trajectory, or stressor-driven decompensation. Plasma MDS-OAβ, while requiring cautious interpretation and broader validation, provides a candidate dynamic axis that can diverge from plaque burden and can be studied in relation to symptom and stress-test trajectories. POD provides a clinically meaningful stress-test phenotype that is supported by human evidence linking delirium with dynamic BBB dysfunction and neuroinflammatory responses. Informed by our recent biomarker-defined MCI/AD cohorts and perioperative observations, this perspective review argues for longitudinal, multi-marker, event-aware models that better match the biology of late life. The practical goal is not to complicate care, but to avoid three common late-life interpretive errors: over-labeling amyloid positivity as the explanation for every symptom, false reassurance from a single negative result, and surrogate confusion between biomarker change and clinical change. A multi-axis, trajectory-based perspective provides a clearer path toward individualized risk stratification and more biologically faithful counseling in older adults.

## Figures and Tables

**Table 1:** Interpretation framework for late-life Alzheimer-spectrum vulnerability using MDS-OAβ, amyloid PET, and postoperative delirium (Refined Version A).

MDS-OAβ	Amyloid PET	POD	Most Plausible Interpretation (Late-Life Framework; With Modifiers)	Recommended Actions/Clinical Use
High (+)	PET+	No	Plaque-positive with elevated oligomerization tendency suggesting an activity-like amyloid milieu without observed stress-test failure in the indexed exposure. In late life, absence of POD may reflect adequate reserve, lower-intensity exposure, or effective prevention, rather than absence of vulnerability.	Longitudinal monitoring of cognition/function; emphasize trajectory (repeat MDS-OAβ when clinically indicated); consider downstream engagement (p-tau/NfL) and comorbidity/reserve profiling; avoid deterministic counseling based on PET alone.
High (+)	PET+	Yes	Convergent high-risk state: plaque burden + activity-like signal + stress-test failure phenotype, consistent with reduced resilience and potentially higher near-term risk after systemic insults.	High-priority risk pathway: delirium-prevention bundle; perioperative optimization; close post-discharge follow-up; multi-axis tracking (MDS-OAβ + downstream markers + neurodegeneration/vascular/frailty measures).
High (+)	PET−	No	Discordant “activity-high/plaque-low” pattern. In very old adults—especially those clinically stable to date—this may reflect heterogeneous biology: (i) early amyloid processes below PET detectability or (ii) non-AD/systemic drivers shaping plasma oligomerization tendency. Interpretable as a risk signal with uncertain substrate, not a diagnosis.	Reassess by trend (repeat MDS-OAβ); triangulate with clinical course and downstream markers; document as uncertain dominant substrate; reconsider imaging only if trajectory warrants.
High (+)	PET−	Yes	Stress-test failure with activity-high signal but no fibrillar plaque detected. POD remains multifactorial; elevated MDS-OAβ may mark a vulnerable milieu, but causal attribution to AD biology is uncertain when PET is negative.	Delirium evaluation/treatment; address triggers aggressively; close cognitive follow-up; broaden vulnerability assessment (frailty/vascular load, inflammatory/metabolic contributors) and consider multi-marker follow-up.
Low (−)	PET+	No	“Silent amyloid” phenotype: fibrillar plaque present with low activity-like signal and no observed stress-test failure. In late life, near-term course may be stable; symptoms (if present) may be dominated by mixed pathology or non-amyloid factors.	Risk-stratify via downstream engagement (p-tau/tau PET if available), neurodegeneration markers, vascular burden and frailty/reserve; counsel probabilistically (substrate ≠ timing).
Low (−)	PET+	Yes	Plaque-positive with stress-test failure but low activity-like signal. Suggests POD driven predominantly by non-amyloid vulnerability axes on a plaque-positive background.	Standard POD work-up/prevention; optimize precipitants; follow cognition after POD; interpret along axes rather than using PET as a single explanatory factor.
Low (−)	PET−	No	Low current AD-spectrum signal with no observed stress-test failure in the assessed window.	Usual care; investigate alternative etiologies if symptoms emerge; repeat biomarkers only if clinically indicated.
Low (−)	PET−	Yes	Event-dominant (non-amyloid) POD phenotype. Particularly in very old and/or low-education individuals, POD may reflect low cognitive reserve/resilience and non-AD mechanisms rather than amyloid-linked activity.	Standard delirium care; optimize triggers; post-event cognitive screening and follow-up (especially if prolonged/recurrent); assess frailty/vascular burden and medication risk profile.

(1) POD(−) means “no delirium observed under the indexed exposure and prevention context,” not “no vulnerability.” (2) Education and age are effect modifiers in late life: lower education may reflect lower cognitive reserve and influence delirium expression and cognitive testing interpretation; advanced age increases the contribution of non-amyloid vulnerability mechanisms. (3) MDS-OAβ is interpreted as an activity-oriented plasma signal (oligomerization tendency) and may be influenced by systemic factors (e.g., inflammatory/metabolic milieu). Therefore, trajectory/pattern is prioritized over a single measurement. (4) This table is a risk-interpretation scaffold rather than a diagnostic classifier; it should not be used for counseling or clinical decision-making without longitudinal confirmation, and clinical judgment remains primary. (5) Late-life discordance commonly reflects mixed pathology; key confounders and differentials to consider across patterns include vascular cognitive impairment, Lewy body disease, depression/sleep disorders, medication effects (e.g., anticholinergics/sedatives), and systemic illness/inflammation.

## Data Availability

No new data were created or analyzed in this study. Data sharing is not applicable to this article.

## Supplementary Materials

The following supporting information can be downloaded at: https://www.mdpi.com/article/10.3390/jpm16030157/s1, Supplementary Box S1: What MDS-OAβ Means and What It Does Not Mean.
