# Supplementary material for: A Multi-Axis Framework for Late-Life Alzheimer’s Disease Interpretation
Source: J Pers Med. 2026 Mar 10;16(3):157. doi: 10.3390/jpm16030157 (PMC13028087; doi:10.3390/jpm16030157)
Supplement: Supplementary file 1 [file jpm-16-00157-s001.zip › jpm-4110461-supplementary.pdf]

Box 1. What MDS-OAβ Means—and What It Does Not Mean

What MDS-OAβ measures (directly)

- A plasma-based oligomerization tendency under standardized assay conditions (a dynamic, aggregation-prone signal in plasma).
- A blood-derived longitudinal signal that can be tracked over time in defined cohorts.

What MDS-OAβ does not measure (directly)

- Brain oligomer burden or synaptic oligomer toxicity.
- Amyloid plaque load or amyloid PET signal.
- A definitive causal mechanism of Alzheimer's disease.

How it can be interpreted (plausible, but not proven)

- May reflect a systemic milieu that modulates aggregation propensity in late life (e.g., inflammatory/metabolic state, proteostasis, vascular/BBB-related influences).
- May co-vary with vulnerability/reserve processes that shape late-life clinical trajectories.

What is empirically observed (association-level evidence)

- In biologically defined cohorts, MDS-OAβ has been reported to show associations with clinically meaningful outcomes/trajectories, and in some settings changes can accompany symptom trajectories (as described in the cited studies).

What remains speculative (mechanistic inference)

- Any one-to-one mapping from plasma MDS-OAβ to brain oligomer dynamics, neurodegeneration, or AD progression is not established.
- Mechanistic interpretations should be treated as testable hypotheses requiring standardized assays, independent replication, and ideally multimodal validation (e.g., imaging/CSF/longitudinal outcomes).
